# Factors Associated with Smoking Cessation and the Receipt of Cessation Services in a Public, Safety-Net Primary Care System

**DOI:** 10.1007/s11606-024-08664-3

**Published:** 2024-02-08

**Authors:** Dian Gu, Henry Rafferty, Maya Vijayaraghavan

**Affiliations:** 1 Center for Tobacco Control Research and Education, University of California, San Francisco, San Francisco, CA USA; 2 Division of General Internal Medicine, San Francisco General Hospital, University of California, San Francisco, San Francisco, CA USA; 3 San Francisco Department of Public Health, San Francisco, CA USA

**Keywords:** associated factors, smoking cessation, cessation service, safety-net setting

## Abstract

**Background:**

Prevalence of smoking is high among patients receiving care in safety-net settings, and there is a need to better understand patient factors associated with smoking cessation and receipt of cessation services.

**Objective:**

To identify patient factors associated with smoking cessation attempts and receipt of cessation counseling and pharmacotherapy in a large safety-net health system.

**Design:**

We conducted a retrospective cohort analysis using EHR data in a safety-net system in San Francisco, CA.

**Participants:**

We included 7384 adult current smokers who had at least three unique primary care encounters with documented smoking status between August 2019 and April 2022.

**Main measures:**

We assessed four outcomes using multivariate generalized estimating equation models: (1) any cessation attempt, indicating a transition in smoking status from “current smoker” to “former smoker”; (2) sustained cessation, defined as transition in smoking status from current smoker to former smokers for two or more consecutive visits; (3) receipt of smoking cessation counseling from healthcare providers; and (4) receipt of pharmacotherapy.

**Key Results:**

Of 7384 current adult smokers, 17.6% had made any cessation attempt, and of those 66.5% had sustained cessation. Most patients (81.1%) received counseling and 41.8% received pharmacotherapy. Factors associated with lower odds of any cessation attempt included being aged 45–64, non-Hispanic black, and experiencing homelessness. The factor associated with lower odds of sustained cessation was being male. Factors associated with lower odds of receiving counseling were being insured by Medicaid or being uninsured. Factors associated with lower odds of receiving pharmacotherapy included speaking languages other than English, being male, and identifying as racial and ethnic minorities.

**Conclusions:**

Health system interventions could close the gap in access to smoking cessation services for unhoused and racial/ethnic minority patients in safety-net settings, thereby increasing cessation among these populations.

## INTRODUCTION

Cigarette smoking prevalence among adults in the United States (US) has been declining, with a prevalence of 20.9% in 2005 to 11.5% in 2021.^1,2^ Despite this improvement, smoking is concentrated among priority populations.^2^ In 2021, smoking prevalence was higher among low-income adults (18.3%) and those uninsured (20.0%) or insured by Medicaid (21.5%) compared to their counterparts. ^2^

Tobacco use remains the leading preventable cause of morbidity and mortality, contributing to over 60% of annual healthcare costs covered by Medicare and Medicaid.^3^ A 1% reduction of smoking prevalence is associated with $2.5 billion in annual Medicaid savings.^4^ Safety-net health systems are the primary source of low or no-cost healthcare for low-income populations. Improving access to smoking cessation care in safety-net settings can reduce tobacco-related health disparities.^5^ Cessation interventions that have included engagement with quitline or in-person tobacco treatment have shown cost savings for safety-net practices^6^ and patients insured by Medicaid.^7^

Within health systems, healthcare providers can help people who smoke to quit by using guideline-recommended 5As for smoking cessation (Ask, Advise, Assess, Assist, and Arrange) and pharmacotherapy.^8^ While most providers Ask, Advise, and Assess for tobacco use, the proportion of providers assisting with quit attempts and arranging for follow-up is much lower.^9^ Digital interventions including reminders in the electronic health record (EHR) have increased provider delivery of 5As.^10^ Moreover, medical teams can use the EHR to receive automatic reminders for tobacco screening,^11^ refer for smoking cessation services (e.g., behavioral counseling and pharmacotherapy),^12^ and monitor receipt of services.^13,14^

Starting in 2009, the federal government put into effect the Meaningful Use criteria, where healthcare systems that had implemented an EHR could receive annual incentive payments from the Centers for Medicare and Medicaid if they met tobacco screening and referral targets.^15^ The implementation of the Meaningful Use EHR guideline for tobacco screening and treatment was associated with an increase in receipt of cessation services among patients who smoked in some health systems.^16–18^

However, only a few studies used the EHR to assess patient characteristics associated with cessation behaviors and receipt of cessation services in safety-net settings.^9,17,19,20^ The results varied depending on the clinical setting and patient population. A study using 2014–2016 EHR data from safety-net clinics in the Oregon Community Health Information Network showed that Medicare patients were less likely to receive counseling and racial and ethnic minorities were less likely to have cessation medications prescribed.^17^ In an EHR-based study from the San Francisco Health Network, a network of safety-net clinics in San Francisco, California, uninsured or patients insured by Medicaid were less likely to receive cessation counseling.^19^ In the same setting, patients who were older, were insured by Medicaid or uninsured, and had limited English proficiency had a lower likelihood of quit attempts.^20^

These studies were done before the COVID-19 pandemic when most cessation services were provided in-person. However, since the pandemic, health systems, including the San Francisco Health Network, have transitioned to providing tobacco treatment using telehealth models of care.^21^ In this study, we extend our prior work^19,20^ by describing previously unexplored patient factors associated with receipt of cessation services, quit attempts, and sustained cessation during the pandemic. Social determinants of health like housing are linked with tobacco use,^22^ and we were able to obtain information on housing status for the present analysis. Our study has two objectives: (1) describe the proportion of smoking cessation attempts, sustained cessation, and receipt of cessation services (counseling and pharmacotherapy) among adult smokers in primary care clinics within the San Francisco Health Network; and (2) identify patient factors associated with those outcomes. We hypothesized that unhoused populations would be less likely to have any cessation and sustained cessation attempts compared to housed populations.

## METHODS

### Study Design and Data Source

We conducted a retrospective cohort study between August 2019 and April 2022 using EHR data from adult patients in 12 primary care clinics within the San Francisco Health Network, a network of primary care clinics that serve San Francisco’s diverse low-income populations. The population includes residents who are low- or very low–income including those experiencing homelessness, and who represent the age, sex, and racial/ethnic diversity of people living in San Francisco. Three clinics were academic primary care practices in a safety-net hospital; the remaining clinics were community-based clinics in San Francisco. At each clinic visit, medical assistants screen patients for current smoking, and document smoking status in EHR as “current smoker,” “former smoker,” or “never smoker.” Medical assistants refer all current smokers to cessation resources (e.g., quitline, or on-site referrals to the medical team), and document referrals within EHR. The medical team, including health care providers and behavioral health care staff, provides tobacco treatment and documents provision of smoking cessation counseling and/or pharmacotherapy during clinical encounters. We extracted data using Structured Query Language (SQL) from the EHR on any patient of the 12 clinics who had at least three unique primary care encounters with documented smoking status between April 2019 and April 2022. The index visit was the first encounter closest to the study’s start date in April 2019. This study was approved by the University of California, San Francisco Committee on Human Research (#18–26,398).

### Ascertainment of Study Cohort

We included adult patients who were aged 18 or over and had at least three unique primary care encounters with documented smoking status. We identified current smokers based on their recorded smoking status at the index visit. Patients could have at minimum three visits and up to nine visits with a documented smoking status throughout the study duration.

### Outcome Variables

The two binary smoking cessation outcomes were (1) any cessation attempt, defined as a transition in smoking status from “current smoker” at the index visit to “former smoker” at any of the subsequent visits, and (2) sustained cessation, defined as transition in smoking status from current smoker at the index visit to former smokers for two or more consecutive visits until the end of the study. The two binary cessation services outcomes for current smokers were (1) receipt of smoking cessation counseling from one or more members of the medical team (i.e., health care provider, medical assistant, or behavioral health staff) at any visit, and (2) receipt of pharmacotherapy including nicotine replacement therapy (NRT) and/or non-NRT at any visit.

### Independent Variables

We assessed independent variables at the patients’ index visit including patient demographics (age [18–44, 45–64, 65 +],^19,20^ sex [male vs. female], race/ethnicity [non-Hispanic White [NHW], Hispanic, non-Hispanic Black [NHB], non-Hispanic Asian [NHA], and non-Hispanic other [NHO]], and primary language), social determinants of health (health insurance and housing status [experiencing homelessness or housed]), and comorbidity burden (number of smoking-related comorbidities [0, 1, 2, ≥ 3] including heart failure, chronic obstructive pulmonary disease, kidney disease, coronary artery disease, depression, diabetes, HIV, hyperlipidemia, and hypertension). We identified comorbidities using ICD-10 codes.^20^ We included intensity of primary care (number of visits during the study period) in the models exploring cessation outcomes.

### Statistical Analysis

First, we used bivariate analysis *χ*^2^ tests to examine the association of independent and cluster variables with each outcome. Then, to determine factors associated with each outcome, we conducted four separate generalized estimating equation (GEE) logistic models, accounting for clustering within the two clinic types (academic vs. community) with an exchangeable structure in each model. We clustered by clinic because patient populations seeking care in academic and community clinics had different demographic characteristics that might be relevant to tobacco use.^20^ The four models estimating the odds of any cessation attempt (model 1), sustained cessation (model 2), receipt of any cessation counseling (model 3), and receipt of pharmacotherapy (model 4) controlled for independent variables from the index visit. Models 1, 3, and 4 were conducted among all current smokers and model 2 was conducted among current smokers who had any cessation attempt. We did not include receipt of cessation services as variables in the models of any cessation attempt or sustained cessation because these services were provided only to patients who were current smokers. Including them in the models would erroneously suggest that current smokers who received cessation services were less likely to quit smoking. To account for time-lapsed between visits, we included average time between visits as a variable in the model for sustained cessation and found no association between sustained cessation and length of time between visits. We chose not to include this variable in the sustained cessation model. All analyses were performed using SAS V.9.4 (SAS Institute, Cary, NC, USA). Estimates were considered to be statistically significant if the two-tailed *p* < 0.05.

## RESULTS

The analytic sample included 7384 adult patients who were current smokers. We excluded 71 patients with incomplete information for the outcome and independent variables. Of the 7384 adult patients who were current smokers, 1302 (17.6%) made any cessation attempt. Of those who made any cessation attempt, 866 (66.5%) made a sustained cessation attempt.

### Sample Characteristics and Factors Associated with Cessation Outcomes

Patients who were younger, identified as Hispanic/Latino, were non-English speaking, had Healthy San Francisco/Healthy Workers coverage, and were housed were more likely to make any cessation attempt (Table 1). In multivariable analysis, being Hispanic (adjusted odds ratio [AOR] 1.53, 95% confidence interval [CI] 1.26–1.86) and number of visits (AOR 1.28, 95%CI 1.24–1.32) were associated with higher odds of making any cessation attempt (Table 2). Factors associated with lower odds of any cessation attempt included older age (45–64 years) (AOR 0.75, 95%CI 0.64–0.87), being NHB (AOR 0.82, 95%CI 0.68–0.97), and experiencing homelessness (AOR 0.53, 95%CI 0.45–0.63). Being male was associated with lower odds of sustained cessation (AOR 0.75, 95%CI 0.58–0.96; Table 2), while number of visits (AOR 1.11, 95%CI 1.04–1.19) was associated with higher odds.

**Table 1:** Characteristics by Any Cessation Attempt Among Current Smokers (*N* = 7384) and Sustained Cessation Among Currents Smokers Who Made Any Cessation Attempt (*N* = 1302)

	Patients with any cessation attempt (*N* = 1302)		Patients with sustained cessation (*N* = 866)	
	*N* (%)	*p*-value	*N* (%)	*p*-value
Demographics				
Age		< .0001		0.5183
18–44	408 (20.2%)		279 (68.4%)	
45–64	632 (15.8%)		411 (65.0%)	
65 +	262 (19.2%)		176 (67.2%)	
Sex		0.0024		0.0227
Female	483 (19.5%)		340 (70.4%)	
Male	819 (16.7%)		526 (64.2%)	
Race/ethnicity		< .0001		0.3253
NHW	305 (15.8%)		193 (63.3%)	
Hispanic	361 (25.3%)		248 (68.7%)	
NHB	305 (14.1%)		195 (63.9%)	
NHA	216 (17.4%)		152 (70.4%)	
NHO	115 (18.4%)		78(67.8%)	
Language		< .0001		0.4121
English	949 (16.4%)		625 (65.9%)	
Other	353 (22.0%)		241 (68.3%)	
Social determinants of health				
Health insurance		0.0070		0.4868
Healthy San Francisco, Healthy Workers	261 (21.3%)		179 (68.6%)	
Medicaid	728 (16.9%)		486 (66.8%)	
Medicare	261 (16.7%)		170 (65.1%)	
Uninsured/self-pay	37 (17.6%)		24 (64.9%)	
Commercial/other	15 (20.3%)		7 (46.7%)	
Housing status		< .0001		0.8584
Experiencing homelessness	182 (10.8%)		120 (65.9%)	
Housed	1120 (19.6%)		746 (66.6%)	
Comorbidity burden				
Number of comorbidities		0.1509		0.4659
0	313 (18.4%)		204 (65.2%)	
1	400 (17.5%)		270 (67.5%)	
2	261 (15.9%)		182 (69.7%)	
3	328 (18.7%)		210 (64.0%)	
Intensity of primary care*				
Number of visits during the study period	7 (3)	< .0001†	7 (3)	0.0220†
Cluster variable				
Primary care center		0.5465		0.0639
Academic	524 (18.0%)		364 (69.5%)	
Community	778 (17.4%)		502 (64.5%)	

*NHW*, non-Hispanic White; *NHB*, non-Hispanic Black; *NHA*, non-Hispanic Asian; *NHO*, non-Hispanic other

*Median (interquartile range). †*p*-value from the Kruskal–Wallis test

**Table 2:** Factors Associated with Cessation Outcomes

	Model 1 outcome: Any cessation attempt		Model 2 outcome: Sustained cessation	
	AOR (95% CI)	*p*-value	AOR (95% CI)	*p*-value
Demographics				
Age				
18–44	Ref			
45–64	0.75 (0.64–0.87)	0.0002	0.85 (0.63–1.14)	0.2805
65 +	0.98 (0.78–1.22)	0.8265	1.00 (0.65–1.53)	0.9911
Sex				
Female	Ref			
Male	0.88 (0.77–1.01)	0.0609	0.75 (0.58–0.96)	0.0223
Race/ethnicity				
NHW	Ref			
Hispanic	1.53 (1.26–1.86)	< .0001	1.23 (0.85–1.77)	0.2722
NHB	0.82 (0.68–0.97)	0.0249	0.96 (0.69–1.35)	0.8282
NHA	0.93 (0.74–1.16)	0.5060	1.43 (0.95–2.16)	0.0891
NHO	1.15 (0.9–1.46)	0.2723	1.21 (0.76–1.91)	0.4293
Language				
English	Ref			
Other	1.07 (0.89–1.29)	0.4604	0.97 (0.69–1.37)	0.8677
Social determinants of health				
Health insurance				
Healthy San Francisco, Healthy Workers	Ref			
Medicaid	0.88 (0.73–1.05)	0.1571	0.93 (0.66–1.3)	0.6644
Medicare	0.84 (0.66–1.06)	0.1359	0.88 (0.57–1.36)	0.5562
Uninsured/self-pay	1.05 (0.71–1.57)	0.7969	0.88 (0.42–1.84)	0.7304
Commercial/other	1.15 (0.63–2.11)	0.6522	0.39 (0.13–1.14)	0.0844
Housing status				
Housed	Ref		Ref	
Experiencing homelessness	0.53 (0.45–0.63)	< .0001	1.12 (0.79–1.58)	0.5290
Comorbidity burden				
Number of comorbidities				
0	Ref			
1	0.96 (0.81–1.13)	0.6011	1.12 (0.81–1.55)	0.4961
2	0.83 (0.69–1.01)	0.0688	1.21 (0.83–1.76)	0.3230
≥ 3	0.94 (0.77–1.14)	0.5289	0.93 (0.64–1.35)	0.6995
Intensity of primary care				
Number of visits during the study period	1.28 (1.24–1.32)	< .0001	1.11 (1.04–1.19)	0.0023

*AOR*, adjusted odds ratio; *95% CI*, 95% confidence interval; *NHW*, non-Hispanic White; *NHB*, non-Hispanic Black; *NHA*, non-Hispanic Asian; *NHO*, non-Hispanic other

### Sample Characteristics and Factors Associated with Receipt of Cessation Services

Most patients who were current smokers received counseling or pharmacotherapy during one or more of their encounters. Among all encounters with counseling, 67% were offered by medical assistants, 0.3% by behavioral health providers, and 33% by healthcare providers (data not shown). Of the 7384 current smokers, 5985 (81.1%) received any counseling and 3088 (41.8%) received pharmacotherapy (Table 3). For receipt of both cessation services, statistically significant differences were found for similar characteristics, except for homelessness status which was not associated with receipt of counseling (Table 3). In multivariable analyses, factors associated with receipt of any counseling and pharmacotherapy were older age (45–64 years) (AOR 1.35, 95%CI 1.17–1.56) and having more comorbidities (Table 4). Factors associated with lower odds of receiving any cessation counseling included having Medicaid (AOR 0.79, 95%CI 0.66–0.95), being uninsured/self-paid (AOR 0.70, 95%CI 0.49–0.99), and having commercial/other insurances (AOR 0.58, 95%CI 0.34–0.98) compared to having “Healthy San Francisco and Healthy Workers.” In contrast, factors associated with higher odds of receiving pharmacotherapy included having Medicare (AOR 1.31, 95%CI 1.12–1.52) and Medicaid (AOR 1.32, 95%CI 1.10–1.60). Being male (AOR 0.87, 95%CI 0.78–0.96), speaking a language other than English (AOR 0.66, 95% CI 0.56–0.77), and being NHA (AOR 0.61, 95%CI 0.51–0.73), NHB (AOR 0.7, 95%CI 0.7–0.90), and NHO (AOR 0.77, 95%CI 0.64–0.93) were factors associated with lower odds of receiving pharmacotherapy.

**Table 3:** Characteristics by the Receipt of Cessation Services Among Current Smokers (*N* = 7384)

	Patients receiving counseling (*N* = 5985)		Patients receiving pharmacotherapy (*N* = 3088)	
	*N* (%)	*p*-value	*N* (%)	*p*-value
Demographics				
Age		< .0001		< .0001
18–44	1498 (74.1%)		759 (37.6%)	
45–64	3331 (83.3%)		1783 (44.6%)	
65 +	1156 (84.7%)		546 (40.0%)	
Sex		0.0028		0.0002
Female	1957 (79.1%)		1110 (44.9%)	
Male	4028 (82.0%)		1978 (40.3%)	
Race/ethnicity		< .0001		< .0001
NHW	1559 (81.0%)		944 (49.0%)	
Hispanic	1075 (75.3%)		530 (37.1%)	
NHB	1767 (81.6%)		980 (45.2%)	
NHA	1088 (87.7%)		366 (29.5%)	
NHO	496 (79.2%)		268 (42.8%)	
Language		0.0185		< .0001
English	4653 (80.5%)		2636 (45.6%)	
Other	1332 (83.1%)		452 (28.2%)	
Social determinants of health				
Health insurance		< .0001		< .0001
Healthy San Francisco, Healthy Workers	1035 (84.4%)		368 (30.0%)	
Medicaid	3413 (79.2%)		1915 (44.4%)	
Medicare	1326 (84.9%)		710 (45.5%)	
Uninsured/self-pay	157 (74.8%)		65 (31.0%)	
Commercial/other	54 (73.0%)		30 (40.5%)	
Housing status		0.0829		0.0003
Experiencing homelessness	1387 (82.5%)		768 (45.7%)	
Housed	4598 (80.6%)		2320 (40.7%)	
Comorbidity burden				
Number of comorbidities		< .0001		< .0001
0	1303 (76.6%)		554 (32.6%)	
1	1836 (80.1%)		897 (39.2%)	
2	1353 (82.7%)		732 (44.7%)	
≥ 3	1493 (85.1%)		905 (51.6%)	
Cluster variable				
Primary care center		< .0001		< .0001
Academic	2155 (73.9%)		1326 (45.5%)	
Community	3830 (85.7%)		1762 (39.4%)	

*NHW*, non-Hispanic White; *NHB*, non-Hispanic Black; *NHA*, non-Hispanic Asian; *NHO*, non-Hispanic other

**Table 4:** Factors Associated with Receipt of Cessation Services

	Model 3 outcome: Counseling		Model 4 outcome: Pharmacotherapy	
	AOR (95% CI)	*p*-value	AOR (95% CI)	*p*-value
Demographics				
Age				
18–44	Ref			
45–64	1.35 (1.17–1.56)	< .0001	1.18 (1.04–1.33)	0.0076
65 +	1.19 (0.96–1.47)	0.1144	0.89 (0.75–1.07)	0.2105
Sex				
Female	Ref			
Male	1.12 (0.99–1.27)	0.0678	0.87 (0.78–0.96)	0.0081
Race/ethnicity				
NHW	Ref			
Hispanic	0.83 (0.69–1.00)	0.0526	0.77 (0.66–0.90)	0.0008
NHB	0.99 (0.85–1.16)	0.9036	0.80 (0.70–0.90)	0.0004
NHA	1.41 (1.12–1.76)	0.0030	0.61 (0.51–0.73)	< .0001
NHO	1.01 (0.81–1.27)	0.8997	0.77 (0.64–0.93)	0.0071
Language				
English	Ref			
Other	1.03 (0.86–1.25)	0.7364	0.66 (0.56–0.77)	< .0001
Social determinants of health				
Health insurance				
Healthy San Francisco, Healthy Workers	Ref			
Medicaid	0.79 (0.66–0.95)	0.0145	1.31 (1.12–1.52)	0.0006
Medicare	0.95 (0.75–1.20)	0.6724	1.32 (1.10–1.60)	0.0035
Uninsured/self-pay	0.70 (0.49–0.99)	0.0461	0.88 (0.63–1.22)	0.4397
Commercial/other	0.58 (0.34–0.98)	0.0426	1.33 (0.81–2.17)	0.2579
Housing status				
Housed	Ref		Ref	
Experiencing homelessness	1.15 (0.99–1.33)	0.0650	1.02 (0.91–1.15)	0.7460
Comorbidity burden				
Number of comorbidities				
0	Ref			
1	1.19 (1.02–1.38)	0.0271	1.27 (1.11–1.45)	0.0005
2	1.34 (1.12–1.60)	0.0013	1.56 (1.35–1.81)	< .0001
≥ 3	1.46 (1.21–1.77)	0.0001	2.14 (1.84–2.49)	< .0001

*AOR*, adjusted odds ratio; * 95% CI*, 95% confidence interval; *NHW*, non-Hispanic White; *NHB*, non-Hispanic Black; *NHA*, non-Hispanic Asian; *NHO*, non-Hispanic other

## DISCUSSION

In this retrospective study of patients seeking primary care in a safety-net health system, we found that 17.6% of patients who smoked attempted to quit smoking, and of those who attempted to quit, 66.5% were able to sustain their quit attempt across two or more visits. Most patients who smoked received cessation counseling, and about half received pharmacotherapy. Quit attempt rates were higher than the spontaneous population-level quitting rate of 5%, but lower than the 30–40% quitting rate observed in 8–12-week clinical trials of behavioral counseling and pharmacotherapy.^23^

In a previous study conducted between 2016 and 2019 in the San Francisco Health Network that examined smoking status across three primary care visits, 26.5% of patients attempted any cessation attempt, and 34% of those who attempted to quit smoking had sustained cessation.^20^ In a related cross-sectional study with a similar population focusing on four safety-net clinics, 17.6% had made any cessation attempt.^19^ The difference in any cessation attempts may be due to unmeasured factors like change in patient population between studies, and the increase in any sustained attempts may be due to following patients over longer periods of time.^19,20^ The present study showed a higher rate of sustained cessation with a stricter measure, a marker of long-term quitting,^24^ and highlighted that most patients were receiving at least one cessation intervention during one or more routine primary care visits. Patients who had a greater number of visits were more likely to make a quit attempt and achieve sustained abstinence.

In the year prior to and during the time of COVID-19 pandemic, the SFHN implemented several quality improvement initiatives to support tobacco treatment efforts,^21^ including a tobacco coordinator who trained clinic staff on how to outreach to patients who smoked, use tobacco registries, implement practice changes in delivering tobacco treatment, and document these interventions in the EHR.^21^ The SFHN also had a robust IT infrastructure that helped transition the health system from an in-person model of healthcare delivery to telehealth model, including for tobacco treatment.^21^ These initiatives may have played a role in increasing patients’ receipt of tobacco treatment, and in turn, their attempts to quit smoking.

Our study highlighted several patient groups that could benefit from expanded and intensive tobacco treatment efforts. People experiencing homelessness had a lower likelihood of any cessation attempts. People experiencing homelessness have high rates of tobacco use, with a prevalence of 70%, but rates of successful cessation are low.^22,25^ In our study, we found that compared to housed individuals, fewer people who were homeless were making any cessation attempts and there was no association with sustained cessation. Our findings suggest that once an attempt is initiated, people experiencing homelessness were no different in sustaining that attempt compared to those who were housed. Therefore, there may be a role for interventions within EHR to increase motivation to quit.^26^

Our study found that male patients were less likely to sustain their cessation compared to women. Men have been shown to have higher nicotine dependence than women,^27^ which is a known barrier to cessation.^28,29^ NHB patients were less likely to make any attempts compared to NHW patients.^30^ Despite smoking at lower rates, NHB patients who smoke have higher levels of nicotine dependence. They are less likely to quit successfully despite making more quit attempts than White smokers.^31^ They may also experience barriers to receiving tobacco treatment,^32^ which we observed in our study and could explain their lower cessation attempt rate. Tobacco retail density tends to be higher in communities where NHB populations reside; the increased availability of tobacco products may influence cessation rates. EHR reminders, although potentially contributing to provider fatigue,^33^ and tobacco registries that systematically address equity gaps may mitigate racial/ethnic disparities in access to tobacco treatment,^21^ may in turn increase cessation attempts.

Patients who were insured by Medicare and Medicaid were more likely to receive cessation pharmacotherapy—a covered benefit—than those covered through Healthy San Francisco where there may have been coverage gaps. In contrast, patients covered through Healthy San Francisco/Healthy Workers were more likely to receive counseling than those on Medicaid. While these findings are consistent with those in our prior study,^19^ they are paradoxical as we expect that Medicaid and Medicare would similarly cover counseling and pharmacotherapy interventions. We speculate that certain omitted variable correlated with one of the cessation services and health insurance coverage affected the direction of this relationship. One of the omitted variables could be providers’ awareness of services covered; most providers may be aware that insurance covers pharmacotherapy but may be less aware that counseling is also a covered benefit. The mechanism of the relationship between health insurance coverage and the receipt of different cessation services warrants further investigation. There may be a need for provider education on the newer recommendations for varenicline^34^ and/or combination NRT for cessation pharmacotherapy.^34^

Racial/ethnic minorities and non-English speaking patients had lower odds of receiving pharmacotherapy. Racial and ethnic minorities tend to have lower rates of pharmacotherapy prescribed than White patients^35^ due to competing priorities among patients and providers, low rates of provider adherence to tobacco treatment guidelines, and implicit bias around adherence to pharmacotherapy among providers of non-White patients.^35^ These barriers may be addressed by creating equity or care gap reminders within the EHR to encourage practice changes around tobacco treatment. Providing education to patients on the benefits of tobacco treatment in a culturally and linguistically concordant manner may also dispel some of the misconceptions around the use of cessation medications among racial/ethnic minority groups.^36,37^

Consistent with our previous studies,^19,20^ medical assistants delivered counseling at higher rates than behavioral health and healthcare providers. This is normative in team-based care settings where the ownership of providing tobacco treatment is distributed among the medical team. However, our results also suggest that while medical assistants may provide the initial referral to treatment, behavioral health and healthcare providers’ support could be enlisted for subpopulation of patients highlighted in this analysis who may benefit from intensive treatments.

Our study had limitations. First, EHR smoking status was based on self-report, potentially resulting in classification errors. Self-report measurements of smoking status have high sensitivity and specificity compared to biochemical validation;^38^ we expect the misclassification rate to be low.^13,20^ Second, nicotine dependence measures were not included in the EHR, highlighting a potential gap in EHR measurements of tobacco use status, which may impact practice changes in prescribing pharmacotherapy. Third, cessation behaviors and service receipt in SFHN may vary from that in other safety-net systems in other geographic areas; therefore, our results may lack external generalizability. Fourth, all the outcomes of interest in the four models were not relatively rare (> 10%), so the AORs in our results might have led to an overestimation of the association strength.^39^ Fifth, because EHR data reflect data captured from clinical encounters, we can only ascertain if and when quitting took place. We are unable to determine whether a lack of transition from current to past smoking was because of lack of motivation to quit or a failed quit attempt. Sixth, we were unable to assess delivery of cessation services between telehealth and in-person encounters, warranting further research to analyze patient characteristics across different methods of delivery.

## CONCLUSION

This study identified subpopulations that could benefit from tailored intensive cessation interventions during primary care. The EHR is a good tool to assess provision of guideline-recommended tobacco treatment to meet performance-based incentive programs for healthcare systems. It is also an effective tool to identify tobacco-related disparities in healthcare settings, allowing healthcare providers and health systems to tailor tobacco treatment to those groups that have the highest gaps in receipt of cessation services. The EHR has the potential to expand collection of social determinants of health metrics, which could provide a deeper understanding of the context of tobacco use and the extent to which tobacco treatment services are reaching disproportionately impacted populations.
